# Comparing the economic burden of disease by physical activity levels among persons with and without disabilities in the Republic of Korea

**DOI:** 10.3389/fpubh.2024.1394378

**Published:** 2024-11-13

**Authors:** SeungCheor Lee, Chang-Mo Oh, Young-il Jung, So-Youn Park, In-Hwan Oh

**Affiliations:** ^1^Department of Preventive Medicine, School of Medicine, Kyung Hee University, Seoul, Republic of Korea; ^2^Department of Environmental Health, Korea National Open University, Seoul, Republic of Korea; ^3^Department of Medical Education and Humanities, School of Medicine, Kyung Hee University, Seoul, Republic of Korea

**Keywords:** amount of physical activity, economic burden, health-related factors, persons with disabilities, Republic of Korea

## Abstract

**Introduction:**

The World Health Organization recommends at least 150 min of physical activity per week for persons with and without disabilities. This study compared the differences in the economic burden of diseases between persons with and without disabilities based on their physical activity. What is known in the field is that physical activity is beneficial to health, but there is a disparity between persons without disabilities and persons with disabilities, and our research aims to increase physical activity among persons with disabilities.

**Methods:**

Economic burden of disease includes service costs such as outpatient, hospitalization, and pharmaceutical expenses for disease treatment, and persons with disabilities are those who have received a disability classification legally registered by the Korean Ministry of Health and Welfare, and there are 15 types of disabilities. For exercise records, we calculated the cost in 2020 for people with exercise records in 2018 so that they can have at least 1 year of exercise records, as health checkups are available from January to December of the year. Physical activity attainment is defined as 150 min or more of physical activity per week based on World Health Organization guidelines.

**Results:**

Calculating the economic burden of disease by physical activity for persons with and without disabilities, both experienced a difference in hospitalization costs owing to fewer hospital days with physical activity achievement, with the difference being greater for persons with disabilities. Regarding disability type, achievers showed a 41.1% difference in total costs for mental class disorders, 16.7% for external bodily function disorders, and 11.1% for internal organ disorders.

**Discussion:**

Physical inactivity has a significant impact on the economic burden of persons with disabilities compared to persons without disabilities, with 11.8% fewer persons without disabilities who were physically active for 150 min or more per week compared to 21.4% fewer persons with disabilities. In terms of healthcare spending, exercise can help keep persons with disabilities healthy. Therefore, there is a need for exercise programs tailored to persons with disabilities to increase physical activity in line with World Health Organization recommendations to reduce health inequalities between persons with and without disabilities.

## 1 Introduction

Physical inactivity has emerged as a global public health concern since 2010 (1). The World Health Organization (WHO) has published physical activity guidelines to promote physical activity globally, recommending at least 150 min of physical activity per week. As of 2020, the same guidelines apply to persons with disabilities (PWDs) (2). In the 2019 Global Burden of Disease Study, the mortality rate associated with low physical activity was 11.1%, and the age-standardized rate of Disability-Adjusted Life Years (DALYs) was 198.4 per 100,000 (3). Physical activity and exercise can improve health and reduce the risk of diabetes, cardiovascular disease, and certain cancers (4), yet approximately 23% of adults worldwide do not engage in adequate physical activity and exercise (5). Among PWDs, a study assessing their participation in physical activity found that 40% did not regularly achieve this goal (6).

Based on the 2012 WHO recommendations, the Korea Health Promotion and Development Agency recommends at least 60 min of moderate and vigorous physical activity daily for children aged 5–17 years, and at least 150 min of aerobic or strength training per week for adults aged 18–64 years. Seniors aged 65 years or older are also recommended to perform at least 150 min of physical activity per week, and if their health condition prevents them from being as physically active as recommended, they are encouraged to exercise as much as possible (7). The global COVID-19 pandemic has led to a decrease in physical activity as people stay home and become more sedentary; therefore, the Korean Ministry of Health and Welfare has been emphasizing walking since April 2021 (7).

The biggest change in the recently revised 2020 WHO Physical Activity Guidelines (November 2020) is a warning that people of all ages, including children and adolescents, should not be “sedentary.” In the previous guidelines, the WHO suggested 10 min as the minimum duration of activity for a single session; however, in the revised guidelines, they changed this to “any amount of physical activity” and emphasized steady movement. “Inactivity” is a leading risk factor for death from non-communicable diseases, and people who are sedentary can have up to a 30% increased risk of premature death compared to those who are active (2). The new revised guidelines recognize that every movement is important in its own right and that it is necessary to understand the importance of being active, such as taking stairs or doing household chores, for good health. They also cited scientific research on the adverse health effects of prolonged sitting (8). The most important thing for health is to achieve the minimum physical activity recommended in the guidelines. Even high-risk individuals, including PWDs, pregnant women, and those with a highly sedentary lifestyle (including those with chronic conditions), should aim for at least 150 min of physical activity per week, with strength training at least twice per week for each body part.

For PWD, increasing physical activity is a useful and low-cost way to stay healthy and has been shown in other research to be effective in preventing disease (9). There are still health inequalities and income disparities between persons with and without disabilities, and our research aims to increase physical activity among PWDs to reduce healthcare spending. In the current situation, in which sedentary lifestyles are expected to increase owing to COVID-19, it is even more important to emphasize physical activity. To our knowledge, to date, no study has measured the economic burden on PWDs in South Korea. Therefore, this study utilized claims data from the National Health Insurance Service (NHIS), which is representative of South Korea, to measure the economic burden of disease among PWDs, and compared persons with and without disabilities and by disability types.

## 2 Materials and methods

### 2.1 Data source and participants

This study used the claims data from the NHIS from 2018 and 2020. We used data on insurance eligibility, premiums, and medical expenditures. We checked demographic variables from insurance eligibility and premium data, and healthcare utilization behaviors, such as the number of outpatient visits and hospitalization days, from the medical expenditure data. The NHIS claims data are cohort-based, allowing for longitudinal research and representativeness of the South Korean population, allowing for reliable analysis of the population of PWDs who have received medical care and health examinations. The NHIS is a social insurance system that covers all South Koreans and enrollment is mandatory, with approximately 97% of the population enrolled as of 2017. The remaining 3% who are not covered by NHIS are eligible for Medical aid, and their claims data are also collected by the NHIS also. The NHIS data contain information such as patient demographics, diagnosis history, consultation history, and prescriptions. Since most adults in South Korea receive health checkups every 2 years, it is possible to conduct research based on these records (10).

**Figure 1:**
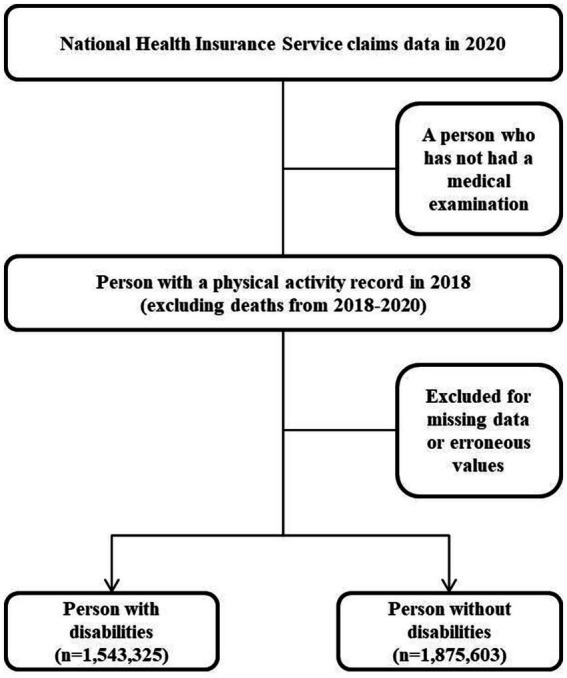
Cohort study design and participant selection criteria.

This study is a cohort study because we measured volume of physical activity in 2018 and analyzed healthcare costs in 2020 by annual base. We excluded the death cases during 2018 to 2020, because we regarded that these death cases are severe disease cases in 2018, so the effect of physical activities could not be measured due to severe illness in registration point. Therefore, the study design for sampling was a cohort study in which participants were first selected for health screening and then followed up with PWDs and persons without disability who were designated as disabled as of 2018 and had a history of physical activity to analyze their healthcare costs in 2020. PWDs were selected from the total number of eligible participants, and non-disabled individuals were randomly selected from the remaining participants as of 2018. Those who died between 2018 and 2020 were excluded from follow-up. Of 3,418,928 people, 1,543,325 PWDs were considered as participants with disability types and severity from the NHIS claims data; 1,875,603 persons without disabilities who met the selection criteria were sampled. The economic burden of disease includes the cost of services such as outpatient visits, hospitalizations, and medications to treat the disease (Figure 1).

### 2.2 Definition of study variables

#### 2.2.1 Persons with disabilities

Participants are PWDs who have been determined to be disabled under the Disability Welfare Act registered with the Ministry of Health and Welfare of the Republic of Korea and are categorized into 15 disability types (11). Based on a doctor’s diagnosis, a person is categorized as having a mild or severe disability based on their medical condition, such as an arm, leg, joint, vision, hearing, etc. Disability types were categorized into legally defined physical disabilities (impairment of external bodily functions, impairment of internal organs) and developmental disabilities. Impairments in external bodily functions include physical disability, brain lesion disorder, hearing impairment, visual impairment, speech impairment, and facial disorder. Disorders of internal organs include heart disorder, kidney disorder, respiratory dysfunction, liver disorder, intestinal fistula/urinary fistula disorder, and epilepsy disorder. Developmental and mental disabilities include intellectual disability, autism spectrum disorder, and mental disorders (12).

#### 2.2.2 Physical activity

Physical activity levels were categorized into two groups according to the WHO guidelines: <150 min of physical activity per week (physical inactivity group) and > 150 min of physical activity per week (physical activity attainment group). Moderate-intensity physical activity was measured using the following question: “During the past week, how many times a week, for how many hours a day, did you engage in moderate-intensity physical activity (e.g., brisk walking, doubles tennis, bicycling, cleaning) for 10 min or more?” High-intensity physical activity was assessed using the following question: “During the past week, for several hours a day, several days a week, did you engage in vigorous levels of physical activity (e.g., running, aerobics, cycling fast) for 10 min or more?” One minute of high-intensity physical activity was considered as 2 min of moderate-intensity physical activity (13). This is a measurement tool created by the NHIS and self-reported by the health checkup participants. For this study, we calculated the economic burden of disease in 2020 for subjects with physical activity records in 2018, assuming they exercised through 2020 (Table 1).

**Table 1:** Economic burden of disease calculations for this study.

Variables			Data source
Direct cost	Medical cost	Insured medical cost	NHIS-claims data^*^ (secondary sources)
		Uninsured medical cost	In-depth data on discharge and injuries NHIS patient medical expenses survey^*^ (secondary sources)
	Non-medical cost	Transportation cost	Korea Health Panel data^*^ (secondary sources)
		Caregiver cost	
Indirect cost	Productivity loss due to morbidity		Korean employment and labor statistics^*^ (secondary sources)

NHIS, National Health Insurance Service.

^*^Health inflation rates: 3%, Unit costs: 2020 USD.

#### 2.2.3 Characteristics

We measured the following variables to find out the characteristics of PWD and non-disabled group: sex, age, health insurance type including Medical aid, income quintile, smoking, drinking, comorbidities, and region of residence. The covariates are socioeconomic characteristics known to affect physical activity (14). Health insurance types of the subscribers were classified into medical aid, regions, and workplace. Income quintiles are categorized into 1, 2, 3, 4, and 5, with higher numbers indicating higher income. In South Korea, everyone has to pay part of their income as insurance premiums except medical aid group. Thus, the higher the quintile, the higher the economic status. The region of residence was classified according to city and province. The cities included Seoul-si, Busan-si, Incheon-si, Gwangju-si, Daegu-si, Daejeon-si, Ulsan-si, and Sejong-si, and the provinces included Gangwon-do, Gyeonggi-do, Chungcheongnam-do, Chungcheongbuk-do, Gyeongsangnam-do, Gyeongsangbuk-do, Jeollanam-do, Jeollabuk-do, and Jeju-do.

Comorbidity was comprehensively evaluated and the Charlson comorbidity index (CCI), which can be applied to various diseases, was used. CCI scores are widely used in medical research because it reflects the severity of comorbidities (14, 15). Comorbidity refers to other diseases the patient has, except the main diagnosis (e.g., diabetes, hypertension). The number of comorbidities was investigated with the question: “Among the following diseases, which diseases have you been diagnosed with, or have you been treated for?”, with the examples including comorbidity, stroke, heart disease (e.g., myocardial infarction, angina pectoris), hypertension, diabetes, dyslipidemia, pulmonary tuberculosis, and other diseases such as cancer. Smoking was measured with the question, “In life, have you ever smoked over five packs of cigarettes (100 pieces)?” Alcohol consumption was measured using the question, “How many times do you drink in a week?”

### 2.3 Statistical analyses

SAS version 9.4 (SAS Institute Inc., Cary, NC, USA) was used for statistical analysis, and the significance level for all statistical tests was set at 5%. Chi-square analyses were conducted to compare the differences in socioeconomic factors between persons with and without disabilities in Table 2 and the achievement of physical activity by disability types in Table 3.

**Table 2:** Characteristics of people with and without disabilities in 2018.

Variables		Person without disabilities		Person with disabilities		*p*-value
		*N*	%	*N*	%	
Sex	Male	1,116,911	59.5	914,774	59.3	<0.001
	Female	758,692	40.5	628,551	40.7	
Age (y)^*^		64.2 ± 14.2		63.9 ± 14.2		<0.001
Health insurance type	Medical aid	48,225	2.6	193,053	12.5	<0.001
	Region (no work)	509,391	27.2	376,100	24.4	
	Workplace	1,317,987	70.3	974,172	63.1	
Region of residence	City	801,238	42.7	599,991	38.9	<0.001
	Province	1,074,365	57.3	943,334	61.1	
Income quintile	Medical aid	48,225	2.6	193,053	12.5	<0.001
	1	289,396	15.4	263,411	17.1	
	2	232,560	12.4	185,197	12.0	
	3	301,914	16.1	231,282	15.0	
	4	387,735	20.7	278,259	18.0	
	5	615,773	32.8	392,123	25.4	
CCI score^*^		3.4 ± 2.7		4.3 ± 3.1		<0.001
Smoking	No	1,556,324	83.0	1,281,749	83.1	0.07
	Yes	319,279	17.0	261,576	16.9	
Drinking	No	1,343,669	71.6	1,210,970	78.5	<0.001
	Yes	531,934	28.4	332,355	21.5	
Physical activity (per week)	<150	1,011,973	54.0	922,575	59.8	<0.001
	≥150	863,630	46.0	620,750	40.2	
Total		1,875,603	100	1,543,325	100	<0.001

CCI, Charlson comorbidity index; N, number; SD, standard deviation.

^*^Mean, standard deviation.

**Table 3:** Compare physical activity attainment per week by disability types in 2018.

Variables		Physical activity (150 min per week)				*p*-value
		<150		≥150		
		*N*	%	*N*	%	
Disability type	Physical disability	501,220	58.9	349,219	41.1	<0.001
	Brain lesion disorder	61,795	63.8	35,132	36.2	
	Visual impairment	92,397	56.5	71,272	43.5	
	Hearing impairment	131,284	58.8	92,113	41.2	
	Speech impairment	5,453	57.8	3,974	42.2	
	Facial disorder	1,041	57.1	782	42.9	
	Kidney disorder	22,449	61.0	14,334	39.0	
	Heart disorder	2,104	59.3	1,443	40.7	
	Liver disorder	3,541	48.5	3,757	51.5	
	Respiratory disorder	3,979	65.8	2,071	34.2	
	Intestinal fistular/urinary fistular disorder	4,067	55.7	3,239	44.3	
	Epilepsy disorder	2,820	63.7	1,604	36.3	
	Intellectual disability	53,681	67.5	25,815	32.5	
	Mental disorder	34,494	71.2	13,972	28.8	
	Autism spectrum disorder	2,250	52.7	2,023	47.3	
Total		922,575	59.8	620,750	40.2	<0.001

N, number.

### 2.4 Economic burden

The economic burden of disease is a measurement tool and can be measured as the sum of direct and indirect costs, as detailed in the papers (15, 16). The economic burden compares the difference in burden between PWDs and persons without disabilities, and the economic burden by disability type. The exchange rate was calculated at 1,180 KRW to the 1 US dollar in 2020 (15). Medical costs of direct costs are categorized into insurance-covered and not covered by insured medical costs. Insurance-covered medical expenses include hospitalization, total outpatient medical expenses, and drugs, while Insurance-uncovered medical expenses are those not covered by insurance. The data source is the NHIS claims data and in-depth data on discharge and injuries from the NHIS patient medical expenses survey from January 2018 to December 2020 (17). The average number of outpatient visits and hospitalizations for persons with and without disabilities due to illness in 2020 was calculated using data from the NHIS. Non-medical costs included transportation and caregiver expenses for hospitalization and outpatient visits, and we used data from the Korea Health Panel. Transportation expenses were measured as follows: “How much was the transportation cost when you used this hospital?” Transportation costs are $25 for inpatient and $1.6 for outpatient on a round-trip basis, multiplied by the number of cases for each disability type. The caregiving cost item was measured as follows: “If you had a paid caregiver during your hospitalization, what was the average cost per day?” The average cost of caregiving is $42.4, multiplied by the number of cases for each disability type. For care expenses, patients under 19 years of age and over 70 years of age at the time of outpatient visit were considered to have a caregiver.

Indirect costs refer to the cost of lost productivity due to illness and morbidity, and include the average cost of lost income per day by age to measure economic losses due to outpatient visits and hospitalizations to treat illness. The loss of productivity due to disease onset was calculated by multiplying the mean daily wage for each age group by the number of days of hospitalization or outpatient visits. Hospitalization days were considered as full day, and outpatient visits were considered as one-third of the day (18). The data source used claims data from the NHIS and South Korean employment and labor statistics to measure lost costs (16). The cost of lost productivity due to illness is the average daily earnings of $50 multiplied by the number of hospital visits for each type of disability.

### 2.5 Ethics statement

This study was conducted after receiving an exemption from review from the Institutional Review Board of Kyung Hee University (no. KHSIRB-23-419). Informed consent was not required because public identity data from the NHIS database were used.

## 3 Results

### 3.1 Characteristics of persons with and without disabilities in 2018

Participants’ physical activity was measured in 2018, and PWDs were more likely than persons without disabilities to engage in less than 150 min of physical activity per week. As of 2020, there were 1,543,325 PWDs in the study group and 1,875,603 persons without disabilities in the control group, totaling 3,418,928 people. As of 2018, PWDs were about 4.8 times more likely than persons without disabilities to be in a medical aid group, with 12.5% of PWDs and 2.6% of persons without disabilities. PWDs had a CCI score of 4.3, compared to 3.4 for persons without disabilities. PWDs were less likely to meet their weekly physical activity goals (46%) than persons without disabilities (40.2%).

Across disability types, all had high rates of less than 150 min of physical activity per week, except those with liver disorders. Persons with mental disabilities had the highest rate of less than 150 min of physical activity (71.2%).

### 3.2 Differences in the economic burden of disease by physical activity level between persons with and without disabilities in 2020

In 2020, the economic burden of disease among PWD was approximately $6,589 ($5,455 direct costs, $1,134 indirect costs) for the low physical activity group and $5,179 ($4,362 direct costs, $817 indirect costs) for the high physical activity group, a difference of 21.4%. The economic burden of disease for people without disabilities was approximately $3,542 ($2,935 direct costs, $607 indirect costs) for the low physical activity group and $3,124 ($2,605 direct costs, $519 indirect costs) for the high physical activity group, a small difference (Table 4).

**Table 4:** Average cost per person according to the amount of physical activity in 2020 (Discount rate: 3%, Unit: $).

Variables			Physical inactivity group		Physical activity attainment group	
			Person with disabilities	Person without disabilities	Person with disabilities	Person without disabilities
Direct cost	Medical cost	Insured medical cost	3,491	1,871	2,841	1,668
		Uninsured medical cost	932	501	767	451
	Non-medical cost	Transportation cost	75	50	66	47
		Caregiver cost	958	513	689	438
Indirect cost	Productivity loss due to morbidity		1,134	607	817	519
Total cost			6,589	3,542	5,179	3,124

### 3.3 Differences in the economic burden of disease by physical activity level by disability type in 2020

In 2020, the economic burden of disease by disability type was the highest for people with kidney dysfunction. The economic burden for people with kidney disability was approximately $34,139 ($31,437 in direct costs and $2,702 in indirect costs) in the low physical activity group and $27,949 ($25,859 in direct costs and $2,091 in indirect costs) in the high physical activity group, indicating a decrease of 18.1%. The largest difference was observed in individuals with autism, who decreased by approximately 46.8 percent in the momentum achievement group (Table 5).

**Table 5:** Average cost per person according to the amount of physical activity by disability type in 2020 (Discount rate: 3%, Unit: 2020 USD).

Variables	Physical inactivity group			Physical activity attainment group		
	Direct cost	Indirect cost	Total cost	Direct cost	Indirect cost	Total cost
Physical disability	4,321	874	5,194	3,604	714	4,318
Brain lesion disorder	7,293	1,717	9,010	5,702	1,234	6,937
Visual impairment	3,917	786	4,703	3,368	652	4,020
Hearing impairment	4,486	950	5,436	3,848	770	4,618
Speech impairment	4,885	1,093	5,978	3,804	786	4,590
Facial disorder	2,920	590	3,510	2,671	575	3,246
Kidney disorder	31,437	2,702	34,139	25,859	2,091	27,949
Heart disorder	9,025	1,081	10,106	8,577	998	9,575
Liver disorder	13,901	864	14,764	13,415	750	14,164
Respiratory disorder	6,762	1,142	7,903	6,633	1,013	7,646
Intestinal fistular/urinary fistular disorder	7,266	1,043	8,309	6,290	886	7,176
Epilepsy disorder	5,209	1,222	6,431	4,087	943	5,030
Intellectual disability	3,736	1,229	4,965	2,403	695	3,098
Mental disorder	11,193	4,443	15,637	7,123	2,435	9,558
Autism spectrum disorder	1,650	497	2,147	907	235	1,142

## 4 Discussion

This research was a cohort study in which participants with physical activity levels in 2018 were selected and followed up to measure healthcare utilization behaviors and healthcare costs in 2020, according to WHO guidelines. They were classified as having less than 150 min of physical activity per week (low) and more than 150 min of physical activity per week (high) (2). We quantified the difference in the economic burden of disease between high- and low physical activity groups.

The relationship between physical activity levels and health status or economic burden has been well described (18). For example, the 2019 Global Burden of Disease study of 204 countries worldwide concluded that high physical activity was associated with disease prevention and reduced premature mortality. For both men and women, mortality and DALYs associated with low physical activity tended to increase with age. And the magnitude of the disease burden of low physical activity varies greatly by age and country, and is closely related to human developmental status; the lower the developmental status, the higher the disease burden (3). Therefore, the results also show that the burden of disease is higher for PWDs than for persons without disabilities.

South Korea has recommended increasing physical activity since 2012 in line with WHO guidelines (19). However, in 2022, only 21% of PWDs engaged in moderate-intensity physical activity and only 18% engaged in vigorous-intensity physical activity (20). For PWDs, increasing physical activity is a useful way to stay healthy in a small amount of time, and many studies have shown its effectiveness in preventing disease (19). High levels of exercise are associated with a variety of health benefits, such as improving cardiorespiratory function and muscle strength, and preventing cardiovascular disease and chronic conditions (e.g., diabetes, hypertension, cancer) (21). Regular participation in exercise can also help maintain independence and prevent health problems secondary to disability (22). Additionally, because PWDs have a higher rate of premature death than persons without disabilities (20), regular physical activity is even more essential for high-risk individuals with disabilities to prevent increased healthcare costs.

Previous studies have analyzed the effects of high-intensity physical activity, moderate-intensity physical activity, and walking on healthcare utilization and healthcare costs in the general population, and our results also show a reduction in healthcare costs. The number of medical visits was reduced by at least 15% and up to 20% for high- and moderate-intensity physical activity, respectively, and by at least 11% and up to 16% for walking, respectively, compared with those who did not have physical activity at all. Out-of-pocket healthcare costs were reduced by at least 13% and up to 20% for high-intensity physical activity, at least 10% and up to 14% for moderate-intensity physical activity, and at least 9% for walking compared to no physical activity (23). Physical activity may be an important health policy tool that can contribute to reducing healthcare utilization and costs on general population.

This study used NHIS claims data to calculate the economic burden of disease in 2020 for PWDs and persons without disabilities and found that the physically inactive group cost PWDs $1,410 more in combined direct and indirect costs and persons without disabilities $418 more than the physically active group. Regarding direct costs, the differences in healthcare costs were 22.6% for PWDs and 12.2% for persons without disabilities, transportation costs were 13.7% for PWDs and 5.7% for persons without disabilities, and care costs were 38.2% for PWDs and 16.8% for persons without disabilities. The indirect cost of lost productivity owing to illness was also reduced by 38.5% for PWDs when they achieved physical activity level, but only by 16.3% for persons without disabilities. This proves that PWDs have a greater cost difference from achieving physical activity than persons without disabilities.

However, the proportion of PWDs who are physically active is low (24), and our results show that all but those with liver disabilities are physically inactive. Among PWDs, those who were physically active had greater differences in healthcare costs over follow-up than non-disabled group, and given the low levels of physical activity, efforts to increase physical activity are needed. The economic burden of disease by physical activity level by disability type showed that outpatient costs were highest for people with kidney disorders in the low physical activity group but decreased in the high physical activity group. Inpatient hospitalization costs were considerably lower for people with intellectual, psychiatric, and autism disorders, and lower costs were associated with achieving physical activity levels. Overall, healthcare costs were highest for people with renal impairment in the low physical activity group, but there was an 18.1% difference in costs in the high physical activity group. Participating in physical activity can improve quality of life and reduce healthcare costs (25).

The differences varied between the disability groups. For example, in terms of total costs, all disability categories showed cost differences with physical activity attainment. Among the disability categories, the mental disability category decreased by 41.1% with physical activity, the external physical dysfunction category decreased by 16.7%, and the internal organ disability category decreased by 11.1%. Our results show that people with mental disabilities have greater cost savings from physical activity, especially compared to existing research that does not differentiate between disability types (23). In particular, in the internal organ disability category, the cost difference was smaller for people with heart, liver, and respiratory disabilities, which could be since they do not see much benefit from physical activity (26). For internal organ disorders, the difference in economic burden from physical activity may be relatively small because internal organ disorders are themselves serious illnesses.

In general, PWDs are less physically active than persons without disabilities (27), and among PWDs, people with mental disorders and intellectual disabilities are particularly less physically active. On average, the income level of persons without disabilities was higher than that of PWDs, and the proportion of PWDs receiving medical aid, which is a basic livelihood security, was about 5 times higher. This suggests that government financial support and the provision of free exercise facilities and vouchers are needed to promote physical activity among low-income households with disabilities, as studies have shown that physical activity levels increase with income (28), suggesting that there is a difference in the cost of investing in exercise based on income level. In addition, PWDs were less likely to be employed than persons without disabilities, which is helpful because other studies have shown that simply having a job promotes more physical activity (29). Comorbidities are higher among PWDs than persons without disabilities, indicating poorer health.

PWDs are known to be more physically inactive as their disability increases in severity (14). For PWDs, there is a need for exercise programs tailored to their severity and social policies to increase access to exercise for PWDs. Through the implementation of these policies, an environment should be created where everyone, regardless of disability, can enjoy the right to health within one system. The main goal of promoting the right to health for PWDs should be to enable them to utilize the resources of their communities to lead healthy and independent lives. The right to access to physical education facilities and the obligation to make reasonable accommodations should be complementary and mutually reinforcing in order to ensure practical access for persons with disabilities, as confirmed in the application of individual countries under the United Nations 11 convention on the rights of persons with disabilities (30).

In a previous study, most participants with disabilities who did not engage in regular physical activity were severely disabled and constrained by a lack of appropriate programs and fear of possible injury during physical activity (6). Therefore, physical activity programs tailored to PWDs should be developed to ensure safety by considering the risks associated with various disability characteristics (31). As the number of PWDs is expected to increase alongside the number of older adult PWDs, healthcare and medical support are expected to emerge as major challenges (32). The proportion of medical expenditure related to disability increases significantly every year (33), but it is expected that health inequalities among PWDs will be reduced if physical activity is actively promoted, considering the effectiveness of physical activity.

Therefore, it is necessary to develop physical activity programs tailored to the type and severity of disability to ensure that PWDs can engage in appropriate levels of physical activity. In addition, healthcare support policies and attention should continue to be directed toward respecting the rights, wishes, and preferences of PWDs and recognizing diversity (30), in line with the 11 convention on the rights of persons with disabilities, to increase physical activity among PWDs through systematic and tailored healthcare policies by disability type. For example, the physical activities available to people with paraplegia are different from those available to people with other disabilities (22), so there is a need to develop tailored exercise programs and provide education to ensure that individuals can be physically active while maintaining the same level of physical activity. Educators should be well trained to deal with PWDs, facilities, families, and schools, and should consider the entire environment.

For PWDs, increasing exercise is a useful way to stay healthy in a less time-consuming way, and many studies have shown its effectiveness in preventing disease (8). High levels of physical activity are associated with a variety of health benefits, such as strengthening of cardiorespiratory function and muscle strength and prevention of cardiovascular disease and chronic diseases (e.g., hypertension, diabetes, and cancer) (4). Regular participation in physical activity can also help maintain independence and prevent health problems secondary to disability (34). By implementing these policies, an environment should be created in which everyone can enjoy the right to health, regardless of disability, within a single system. The basic goal of promoting the right to health of PWDs should be to support them so that they can lead healthy and independent lives by utilizing resources in their own communities.

This study has a few limitations. PWDs need someone to help them with physical activity, and further cost effectiveness analysis of these physical activity program would provide a clearer estimate of the benefits of activity. Also, the level of physical activity could be influenced by the degree of disability; therefore, healthcare costs or the economic burden of disease may be higher in the less active group. In addition, since only costs were considered, characteristics such as sex and age of the physically active and non-physically active groups were not adjusted. Additionally, the amount of alcohol consumed was not considered in the drinking measure and age was not considered in the smoking measure. Finally, we only included people who participated in the health screening. Therefore groups with such a high degree of disability that they did not receive a medical examination were excluded from the analysis.

## 5 Conclusion

PWDs have a higher economic burden than persons without disabilities. To address this, it is important to promote healthy physical activity and prevent chronic diseases among PWDs. In order to determine the potential size of the disease burden that can be reduced for the promotion of physical activity, we tried to determine the difference in the disease burden between the physical activity achievement group and the non-achieving group, ad also tried to identify this by disability type. As a result, the difference in economic burden depending on whether or not the level of physical activity is achieved was greater for the PWDs than for the non-disabled, and the physical activity achievement group have only 78% of the economic cost compared to non-attainment group. Therefore, future physical activity policies need to be aligned with WHO recommendations using the current findings to address health inequalities of PWDs. In accordance with the 11 convention on the rights of persons with disabilities, physical activity policies, promotion plans and programs should be facilitated at the national, regional and international levels to further equalize opportunities for PWDs.

## Data Availability

The datasets presented in this study can be found in online repositories. The names of the repository/repositories and accession number(s) can be found in the article/supplementary material.
